# Evaluation of HPV and Related Cancer Awareness and Vaccination Attitudes Among Patients with Anogenital Warts: a Survey-Based Study

**DOI:** 10.1007/s10900-025-01444-y

**Published:** 2025-02-02

**Authors:** Berna Solak, Mustafa Arslan

**Affiliations:** Department of Dermatology, School of Medicine, Sakarya University, Sakarya, Turkey

**Keywords:** Anogenital warts, Awareness, Cervical cancer, Human papillomavirus, Public health strategies, Vaccination attitudes

## Abstract

We aimed to evaluate awareness of HPV and its associated cancers, attitudes toward HPV vaccination, and vaccination rates in individuals with anogenital warts. This cross-sectional study was conducted at Sakarya University Training and Research Hospital using a questionnaire completed by individuals diagnosed with anogenital warts. A total of 105 respondents were included in the study, comprising 80 males (76.2%) and 25 females (23.8%). The mean age of participants was 34.7 ± 11.2 years. HPV awareness was 70.5%, while cervical cancer awareness was 38.1%. Women demonstrated significantly higher levels of HPV and cervical cancer awareness, as well as knowledge of Pap smear testing, compared to men. Women were also significantly more likely than men to express willingness to vaccinate their children against HPV (84.0% vs. 58.8%, *p* = 0.039). Higher education levels were associated with increased awareness of HPV, HPV vaccination, and willingness to vaccinate children. Physicians were the primary source of HPV-related information across the cohort. HPV vaccine awareness was 73.3%, but the overall vaccination rate was only 10.5%, with women showing significantly higher vaccination rates than men (24% vs. 6.2%, *p* = 0.021). The most commonly reported barriers to vaccination were cost (60%) and lack of information (45.7%). None of the participants had vaccinated their children. This study highlights that awareness of HPV and its vaccination is associated with gender and education level but does not translate into higher vaccination rates. Efforts should focus on targeting men and individuals with lower educational attainment by strengthening physicians’ roles in public education. Incorporating HPV vaccination into national programs and implementing culturally tailored campaigns may effectively improve vaccination rates.

## Introduction

Human papillomavirus (HPV) is a DNA virus with over 200 types that infects the skin and mucosal surfaces [1]. It is among the most common sexually transmitted infections globally, causing conditions ranging from anogenital warts to cancers. High-risk HPV types are major contributors to cancers of the cervix, vulva, vagina, anus, penis, and oropharynx [1–3]. HPV, attributed to approximately 690,000 new cancer cases globally in 2018, represents a significant public health concern [4].

Prevention of HPV infections is achievable through risk-reducing measures and vaccination. While condom use partially reduces HPV transmission, its effectiveness is limited due to potential exposure from unprotected areas. Vaccines containing virus-like particles made of capsid proteins, provide effective protection against high-risk HPV types [1]. Given the prevalence of HPV infections and the substantial burden of associated conditions on healthcare systems, raising awareness and implementing prevention strategies are essential.

To our knowledge, this may be among the first studies that have simultaneously evaluated awareness of HPV, its associated cancers, and vaccination attitudes among individuals with anogenital warts. This study aims to assess awareness of HPV and related cancers (e.g., cervical, anal, and penile cancer), attitudes toward HPV vaccination, and vaccination rates in this population. The findings are expected to provide a scientific basis for awareness campaigns and strategies to enhance vaccination rates.

## Methods

### Study Design and Setting

This cross-sectional study was conducted at the Dermatology Outpatient Clinic of Sakarya University Training and Research Hospital, involving patients diagnosed with anogenital warts. Data collection took place between July 1 and November 30, 2024. Ethical approval was obtained from the Sakarya University Scientific Research Ethics Committee (Approval No: E-43012747-050.04-421216-155).

### Participant Selection and Data Collection

Participants were included in the study on a voluntary basis. During the study period, all patients diagnosed with anogenital warts at the clinic were provided with detailed information about the study’s purpose and methods. Those who consented to participate were enrolled. Data were collected through self-administered questionnaires completed by participants in a private room. The questionnaires did not include identifying information, and data were collected anonymously.

Participants eligible for inclusion were adults aged 18 years or older with a diagnosis of anogenital warts, capable of providing informed consent. Of the 105 participants included in the study, 80 were males and 25 were females. Exclusion criteria included individuals who were unable or unwilling to answer the questions, as well as questionnaires containing incomplete or invalid data. Exclusion criteria included individuals who were unable or unwilling to answer the questions, those with incomplete or invalid questionnaire data, or those with concurrent HIV, syphilis, or other sexually transmitted infections.

### Questionnaire

The questionnaire consisted of 26 items assessing demographic data, awareness of HPV, cervical cancer, Pap smear testing, the association of HPV with other cancers, HPV vaccination, and attitudes toward vaccination. We developed based on insights from previous HPV awareness studies conducted in various populations [5–7]. After a pilot study, the questions were refined to ensure clarity for the target population. Awareness was assessed using questions such as “Have you ever heard of this?” Information about HPV-related cancers was evaluated using the phrasing “Could it cause this?”. Most questions were structured as “Yes” or “No” responses, with an open-ended option provided only under “Reason for Vaccine Hesitancy.”

### Statistical Analysis

Sample size was calculated using the prevalence value (154/100,000) reported by Ozgul et al. [8] in Turkey. Based on this, with a power of 80% and an effect size of 0.01, the minimum required sample size was determined to be 121. Data were analyzed using SPSS v.20 (SPSS Inc., Chicago, IL, USA). Categorical variables were presented as frequencies and percentages, and group comparisons were performed using Chi-square and Fisher’s Exact tests. Normality of the age variable was assessed with the Kolmogorov-Smirnov test and Q-Q plots, and results were expressed as mean ± standard deviation. Independent Samples t-Test and One-Way ANOVA were used for age comparisons between groups. Bonferroni correction was applied for post-hoc analyses. Statistical significance was set at *p* < 0.05.

## Results

### Patient Characteristics and Knowledge About HPV

A total of 126 survey forms were collected at the end of the study, of which 105 fully completed forms were included in the analysis. Of these participants, 80 were male (76.2%) and 25 were female (23.8%). The mean age of participants was 34.7 ± 11.2 years, and 76.2% were male. Educational levels of the participants were as follows: 10 (9.5%) were Literate/Primary, 44 (41.9%) had completed Secondary/High School, and 51 (48.6%) were University/Postgraduate graduates.

Overall, HPV awareness was 70.5%, while cervical cancer awareness was 38.1%. No significant differences were observed between males and females in terms of demographics, awareness of HPV, or knowledge about anogenital warts. Detailed comparisons of knowledge regarding HPV and associated conditions are presented in Table 1, while Table 2 provides data on awareness and preferences regarding HPV vaccination.

Awareness of HPV and anogenital warts increased significantly with higher educational attainment. Among university graduates, 86.3% were aware of HPV, compared to only 10% in the lowest educational group (*p* < 0.001). Similarly, the recognition of HPV as a cause of anogenital warts was significantly higher among participants with higher education levels (*p* < 0.001, Table 3).

### Knowledge about HPV-related Conditions

Among women, 40% reported having undergone at least one Pap smear test. While 36% took no preventive measures against cervical cancer, the most common strategy was regular Pap smear screening (32%). Other measures included HPV vaccination (24%), limiting the number of sexual partners (24%), and condom use (16%).(Participants could select multiple options for this question.)

Awareness that HPV causes cervical cancer (64%) and that the HPV vaccine is effective in its prevention (72%) was significantly higher among women compared to men (32.5% and 42.5%, respectively; *p* = 0.010 and *p* = 0.019, respectively) (Table 1). Awareness that HPV can cause cancer in men increased significantly with higher education levels (*p* < 0.001). No participants in the lowest education group had this knowledge, compared to 59.1% in the secondary education group and 72.5% among university graduates. Similarly, awareness that HPV can cause penile cancer varied significantly by education level (*p* = 0.002). While 60.8% of university graduates were aware, no participants in the primary education group had this knowledge (Table 3).

### Knowledge and Preferences on HPV Vaccine

In the entire cohort, HPV vaccine awareness was 73.3%, but the vaccination rate was only 10.5%. Among women, 24% had received the vaccine compared to 6.2% of men (*p* = 0.021). Of those vaccinated, 18.2% chose the quadrivalent vaccine, while 81.8% received the nonavalent vaccine.

Sources of information on the HPV vaccine included physicians (49.4%), internet/news (45.5%), and partners/friends (5.2%). Sources of HPV vaccine information varied by education level. Participants with basic education relied equally on physicians and partners/friends, those with secondary education primarily consulted physicians (63.3%), and university graduates favored media sources (57.8%) (Table 4).

Among unvaccinated participants, 37.2% indicated they were considering HPV vaccination. Reasons for vaccine hesitancy (with multiple responses allowed) included cost (60.0%), perceived harmfulness (45.7%), lack of knowledge (45.7%), and other reasons (12.8%). No statistically significant differences in these reasons were observed based on gender or education level (Tables 2 and 4). Other reasons cited included partner/spouse disapproval, lack of time, procrastination, and forgetfulness.

Overall, 64.8% of participants expressed willingness to vaccinate their children. Participants who answered “yes” to vaccinating their children against HPV were younger (mean age 32.7 ± 9.3 years) than those who answered “no” (38.3 ± 13.6 years) (*p* = 0.031). Among those willing, 54.4% were aware that HPV causes anogenital warts, compared to 13.5% of those unwilling, a statistically significant difference (*p* < 0.001). Additionally, 90.9% of vaccinated participants expressed willingness to vaccinate their children, compared to 61.7% of unvaccinated participants, although this difference was not statistically significant (*p* = 0.092).

A significantly higher proportion of women (84%) than men (58.8%) expressed willingness to vaccinate their children against HPV (*p* = 0.030). This intention also varied by education level, increasing significantly with higher education, from 30% in the primary education group to 72.5% among university graduates (*p* = 0.048). Notably, none of the participants had vaccinated their children.

**Table 1:** Comparison of demographic characteristics, knowledge about HPV and HPV-related conditions by gender

Survey item	Female (*n* = 25)	Male (*n* = 80)	*p*-value
Age (years)	33.7 ± 10.2	35.0 ± 11.6	0.587
**Education Level**			
Literate/Primary Education	4 (16.0%)	6 (7.5%)	0.391
Secondary/High School (graduate)	9 (36.0%)	35 (43.8%)	
University/Postgraduate (student/graduate)	12 (48.0%)	39 (48.8%)	
**Knowledge about HPV**			
Awareness of HPV	19 (76.0%)	55 (68.8%)	0.658
Knowledge that HPV can cause anogenital warts	18 (72.0%)	52 (65.0%)	0.685
Knowledge that condom use may prevent HPV infection	17 (68.0%)	47 (58.8%)	0.553
**Knowledge about Cervical Cancer**			
Awareness of cervical cancer	19 (76.0%)	21 (26.2%)	< 0.001
Knowledge that HPV can lead to cervical cancer	16 (64.0%)	26 (32.5%)	0.01
Knowledge of the Pap smear test	21 (84.0%)	15 (18.8%)	< 0.001
Has undergone a Pap smear test (only women)	10 (40.0%)	-	-
Knowledge of the importance of the Pap smear test in cervical cancer	17 (68.0%)	24 (30.0%)	0.002
Interest in further information about cervical cancer	25 (100.0%)	69 (86.2%)	0.063
**Knowledge about Other Cancers**			
Knowledge that HPV can cause cancer in men	13 (52.0%)	50 (62.5%)	0.483
Knowledge that HPV can cause oropharyngeal cancer	5 (20.0%)	33 (41.2%)	0.091
Knowledge that HPV can lead to penile cancer	10 (40.0%)	43 (53.8%)	0.331
Knowledge that HPV can lead to anal cancer	12 (50.0%)	41 (51.2%)	> 0.999

**Table 2:** Comparison of knowledge and preferences on HPV vaccine by gender

Survey Item	Female (*n* = 25)	Male (*n* = 80)	*p*-value
**Knowledge about the HPV Vaccine**			
Awareness of the HPV vaccine	22 (88.0%)	55 (68.8%)	0.101
Source of information on the HPV vaccine (among those aware)			
- From physician	11 (50.0%)	27 (49.1%)	
- From partner/friends	3 (13.6%)	1 (1.8%)	0.112
- From internet, TV, or other media sources	8 (36.4%)	27 (49.1%)	
Knowledge of the HPV vaccine’s role in preventing cervical cancer	18 (72.0%)	34 (42.5%)	0.019
Knowledge of the number of doses required for HPV vaccination	11 (44.0%)	25 (31.2%)	0.328
Has received the HPV vaccine	6 (24.0%)	5 (6.2%)	0.021
Reason for Vaccine Hesitancy^**^			
- Perceived harmfulness of the vaccine	10 (52.6%)	33 (44.0%)	0.608
- Cost	11 (57.9%)	46 (61.3%)	0.798
- Lack of knowledge	12 (63.2%)	31 (41.3%)	0.122
- Other reasons	4 (21.1%)	8 (10.7%)	0.253
Willingness to receive the HPV vaccine (among those unvaccinated)			
- Willing to be vaccinated	13 (68.4%	41 (54.7%)	0.311
- Not willing to be vaccinated	6 (31.6%)	34 (45.3%)	
Vaccine preference among those willing to be vaccinated			
- Quadrivalent HPV vaccine	5 (38.5%)	10 (24.4%)	0.478
- Nonavalent HPV vaccine	8 (61.5%)	31 (75.6%)	
Willingness to vaccinate children	21 (84.0%)	47 (58.8%)	0.039

^**^ Participants were allowed to select more than one option

**Table 3:** Comparison of demographic characteristics, knowledge about HPV and HPV-related conditions by education level

Survey Item	Literate/Primary (*n* = 10)	Secondary/High School (*n* = 44)	University/	*p*-value
			Postgraduate (*n* = 51)	
Age (years)	49.9 ± 14.7	37.1 ± 11.1	29.6 ± 6.3	< 0.001^*^
**Gender**				
Female	4 (40.0%)	9 (20.5%)	12 (23.5%)	0.390
Male	6 (60.0%)	35 (79.5%)	39 (76.5%)	
**Knowledge about HPV**				
Awareness of HPV	1 (10.0%)	29 (65.9%)	44 (86.3%)	< 0.001^ɸ^
Knowledge that HPV can cause anogenital warts	1 (10.0%)	29 (65.9%)	40 (78.4%)	< 0.001^ɸ^
Knowledge that condom use may prevent HPV infection	3 (30.0%)	30 (68.2%)	31 (60.8%)	0.079
**Knowledge about Cervical Cancer**				
Awareness of cervical cancer	2 (20.0%)	16 (36.4%)	22 (43.1%)	0.408
Knowledge that HPV can lead to cervical cancer	1 (10.0%)	17 (38.6%)	24 (47.1%)	0.106
Knowledge of the Pap smear test	2 (20.0%)	15 (34.1%)	19 (37.3%)	0.655
Has undergone a Pap smear test	2 (50.0%)	6 (75.0%)	2 (20.0%)	0.095
Knowledge of the importance of the Pap smear test in cervical cancer	3 (30.0%)	15 (34.1%)	23 (45.1%)	0.505
Interest in further information about cervical cancer	8 (80.0%)	38 (86.4%)	48 (94.1%)	0.210
**Knowledge about Other Cancers**				
Knowledge that HPV can cause cancer in men	0	26 (59.1%)	37 (72.5%)	< 0.001^a^
Knowledge that HPV can cause oropharyngeal cancer	1 (10.0%)	15 (34.1%)	22 (43.1%)	0.131
Knowledge that HPV can lead to penile cancer	0	22 (50.0%)	31 (60.8%)	0.002^a^
Knowledge that HPV can lead to anal cancer	2 (20.0%)	21 (47.7%)	30 (60.0%)	0.054

Subgroup comparisons (after Bonferroni corrections): * Significant difference between all three groups. ɸ Difference between the Literate/Primary Education group and both the Secondary/High School and University/Postgraduate groups. α Difference between the Literate/Primary Education group and both the Secondary/High School and University/Postgraduate groups

**Table 4:** Comparison of knowledge and preferences on HPV vaccine by education level

Survey item	Literate/Primary education (*n* = 10)	Secondary/High School (*n* = 44)	University/	*p*-value
			Postgraduate (*n* = 51)	
**Knowledge about the HPV Vaccine**				
Awareness of the HPV vaccine	2 (20.0%)	30 (68.2%)	45 (88.2%)	< 0.001^ɸ^
Source of information on the HPV vaccine (among those aware)				
- From physician	1 (50.0%)	19 (63.3%)	18 (40.0%)	0.011^l^
- From partner/friends	1 (50.0%)	2 (6.7%)	1 (2.2%)	
- From internet, TV, or other media sources	0	9 (30.0%)	26 (57.8%)	
Knowledge of the HPV vaccine’s role in preventing cervical cancer	2 (20.0%)	22 (50.0%)	28 (54.9%)	0.130
Knowledge of the number of doses required for HPV vaccination	1 (10.0%)	14 (31.8%)	21 (41.2%)	0.165
Has received the HPV vaccine	0	5 (11.4%)	6 (11.8%)	0.797
Reason for Vaccine Hesitancy**				
- Perceived harmfulness of the vaccine	6 (60.0%)	20 (51.3%)	17 (37.8%)	0.316
- Cost	5 (50.0%)	23 (59.0%)	29 (64.4%)	0.652
- Lack of knowledge	5 (50.0%)	20 (51.3%)	18 (40.0%)	0.610
- Other reasons	3 (30.0%)	4 (10.3%)	5 (11.1%)	0.250
Willingness to receive the HPV vaccine (among those unvaccinated)				
- Willing to be vaccinated	3 (30.0%)	21 (53.8%)	30 (66.7%)	0.098
- Not willing to be vaccinated	7 (70.0%)	18 (46.2%)	15 (33.3%)	
Vaccine preference among those willing to be vaccinated				
- Quadrivalent HPV vaccine	1 (33.3%)	8 (38.1%)	6 (20.0%)	0.358
- Nonavalent HPV vaccine	2 (66.7%)	13 (61.9%)	24 (80.0%)	
Willingness to vaccinate children	3 (30.0%)	28 (63.6%)	37 (72.5%)	0.048^c^

^**^ Participants were allowed to select more than one option. Subgroup comparisons (after Bonferroni corrections): ɸ Difference between the Literate/Primary Education group and both the Secondary/High School and University/Postgraduate groups. λ Difference between Literate/Primary Education and University/Postgraduate groups for responses from partner/friend. χ Difference between the Literate/Primary Education and University/Postgraduate groups

## Discussion

The key findings of our study are as follows: Awareness of cervical cancer, the relationship between HPV and cervical cancer, the role and importance of Pap smear testing, knowledge of the preventive role of the HPV vaccine, vaccination uptake, and willingness to vaccinate children were all significantly higher among women than men. Awareness of HPV and its role in causing anogenital warts, cancer in men, and penile cancer increased significantly with higher education levels. Similarly, awareness of the HPV vaccine and willingness to vaccinate children also rose with increasing education. Sources of information about the HPV vaccine varied by education level. Participants with lower education levels were more likely to rely on partners or friends, whereas university graduates predominantly used media sources (57.8%), and those with basic literacy or primary education primarily consulted physicians. Additionally, participants who were aware that HPV causes anogenital warts were significantly more likely to express willingness to vaccinate their children. Notably, none of the participants had yet vaccinated their children against HPV.

Studies have generally reported higher HPV awareness among women compared to men [9, 10]. However, Stephens et al. [11] found similar awareness levels for both HPV and its vaccine among genders, at around 50%. In our study, 76.0% of women and 68.8% of men were aware of HPV, and knowledge of its link to anogenital warts was 72.0% in women and 65.0% in men, with no significant gender differences. Honnavar et al. [5] noted that while 91.6% of women in their study were aware of HPV, only 12.8% had been vaccinated. In contrast, HPV awareness among women in our study was lower (76.0%), but vaccination rates were higher (24%), suggesting that vaccination rates depend not only on awareness but also on factors like healthcare access, societal perceptions, and health policies. Furthermore, HPV and vaccine awareness have been shown to increase with higher educational levels [9–11]. For instance, Wheldon et al. [9], reported a greater rise in HPV awareness among college graduates compared to high school graduates. Consistent with these findings, our study showed significantly higher HPV awareness and knowledge of its link to anogenital warts among university graduates, with the lowest levels observed among individuals with only primary education or literacy.

Wheldon et al. [9] reported that a large proportion of participants were aware of the association between HPV and cervical cancer, with this awareness increasing over time (from 78 to 81.5%). Similarly, Stephens et al. [11], found that 50–80% of the general population recognized HPV as a cause of cervical cancer, with variations based on race and education. Interestingly, Arechkik et al. [12] demonstrated in a 2023 study of HIV-positive women that, while cervical cancer awareness was high, knowledge of its link to HPV was lacking. Likewise, Belglaiaa et al. [13] observed that only 20.9% of HIV-positive women were aware of cervical cancer, and none associated it with HPV. In our study, cervical cancer awareness was higher among women (76.0%) than men, and 64.0% of women recognized its link to HPV. Participants with anogenital warts demonstrated significantly greater awareness compared to HIV-positive women in previous studies. Notably, in our study, men’s awareness of cervical cancer and its association with HPV was significantly lower than that of women. Interestingly, our study found no significant difference in the willingness to learn more about cervical cancer between women and men (100.0% vs. 86.2%), highlighting the importance of addressing both genders in awareness efforts.

In the study by Stephens et al. [11], cancer awareness varied by education level, with over 80% of university graduates recognizing the link between HPV and cervical cancer, compared to approximately 50% among those with lower education levels. In our study, cervical cancer awareness ranged from 20 to 43%, and awareness of HPV as a cause of cervical cancer ranged from 10 to 47%, both highest among university graduates but without statistically significant differences. Compared to the previously mentioned study, cervical cancer awareness was lower across all education levels in our cohort, despite consisting of HPV-positive patients with anogenital warts. Studies report that approximately 30% of men and women are aware of HPV’s link to anal, oral, and penile cancers [6, 9, 11]. In our study, this proportion was slightly higher, with about half of the participants knowing HPV could cause cancer in men, particularly penile and anal cancers. Awareness was highest among university graduates (72.5%). The higher awareness may stem from participants’ experience with HPV-related anogenital warts, increasing their exposure to relevant information.

A study on cervical cancer and HPV awareness among women in Antigua and Barbuda [5], reported that 87.8% of women had undergone a Pap smear in the past decade, with higher awareness among those with higher education, attributed to physician-led health education, free testing, and the influence of the internet and social media. In HIV-positive women, Belglaiaa et al. [13], found that the most commonly cited preventive methods against cervical cancer were condom use (10.4%) and regular Pap smear testing (9.6%). In our study, 40% of women reported having undergone at least one Pap smear. Compared to HIV-positive women in previous studies, our cohort of women with anogenital warts had a significantly higher Pap smear rate, likely due to free screening provided by Turkey’s Cancer Early Diagnosis, Screening, and Training Centers (KETEM). However, 36% of women in our study reported taking no preventive measures, while the most common preventive actions included Pap smear testing (32%), HPV vaccination (24%), limiting the number of sexual partners (24%), and condom use (16%).

In our study, 88% of women and 69% of men were aware of the HPV vaccine. Awareness of its role in preventing cervical cancer was significantly higher in women than men. Among those not vaccinated, more women expressed willingness to be vaccinated, but this difference was not statistically significant. HPV vaccination rates were significantly higher in women (24%) than men. Vaccine awareness increased with education, from primary school graduates to university graduates. However, vaccination rates and willingness to be vaccinated showed no significant differences across education levels, suggesting that access, cost, or cultural barriers may play a role beyond awareness.

A systematic review of community-based studies in Turkey (2019) revealed wide variations in HPV and HPV vaccine awareness, with HPV awareness ranging from 3.8 to 57.0% and HPV vaccine awareness from 2.2 to 74.7% [14]. Vaccination rates reported in the systematic review were low (0.3–6.0%), while willingness to be vaccinated varied between 6.3% and 69.0%. Common barriers included lack of knowledge, concerns about side effects, and cost, reflecting regional and cultural differences. In our study, unvaccinated participants most frequently cited cost, misconceptions about vaccine safety, and lack of information as reasons for not getting vaccinated. While lower education levels were associated with safety concerns and higher education levels with cost, these differences were not statistically significant. These findings highlight the need to address both perceptual and structural barriers to improve vaccination rates. Additionally, the COVID-19 pandemic and related misinformation may have exacerbated vaccine hesitancy. Physicians were identified as the primary source of HPV vaccine information in our study, followed by online sources, suggesting that awareness efforts should be tailored to different educational and informational access levels. Notably, all participating women and 86.2% of men expressed a desire for more information about cervical cancer and HPV, indicating a persistent knowledge gap even among those with anogenital warts.

Wang et al. [6], found that in China, awareness and willingness to vaccinate were higher among university-educated parents, but actual vaccination rates were higher among less-educated parents, attributed to preferences for local vaccines and accessibility issues with imported ones. Similarly, a study in Saudi Arabia [7], showed that while about half of university students were aware of HPV and cervical cancer, only 36.2% knew of the HPV vaccine, and 10% were vaccinated. In our study, while women exhibited a slightly higher willingness to receive the HPV vaccine, this difference was not statistically significant. However, women showed a significantly greater inclination to vaccinate their children, a trend that similarly increased with higher levels of education. Additionally, 90.9% of vaccinated participants and 61.7% of unvaccinated participants expressed a willingness to vaccinate their children. Despite these high rates of willingness, none had taken action to vaccinate their children, underscoring a critical gap between awareness and behavior and the urgent need to address barriers to HPV vaccination.

This study has several limitations worth noting. First, the survey relied on self-reported data. Second, the single-center design may limit the generalizability of the findings. Nevertheless, as the first awareness study conducted in a population with anogenital warts, it provides a valuable insigths into an important health topic.

In conclusion, this study demonstrates that awareness and attitudes toward HPV, HPV-related conditions, and the HPV vaccine are influenced by gender and education level. Notably, negative vaccination attitudes were more prominent among men and individuals with lower education levels. Although awareness was higher among women and those with higher education levels, this did not sufficiently translate into higher vaccination rates. The significant role of physicians as a primary source of information highlights the need to intensify awareness efforts through healthcare channels. Including the HPV vaccine in national immunization programs, along with culturally and socially tailored campaigns supported by public figures and educational initiatives in schools, may help improve vaccine acceptance.

## Data Availability

The data that support the findings of this study are available from the corresponding author, upon reasonable request.
